# Cardiorespiratory Fitness as a Correlate of Cardiovascular, Anthropometric, and Physical Risk Factors: Using the Ruffier Test as a Template

**DOI:** 10.1155/2020/3407345

**Published:** 2020-09-08

**Authors:** Khalid A. Alahmari, Kanagaraj Rengaramanujam, Ravi Shankar Reddy, Paul Silvian Samuel, Venkata Nagaraj Kakaraparthi, Irshad Ahmad, Jaya Shanker Tedla

**Affiliations:** Department of Medical Rehabilitation Sciences, College of Applied Medical Sciences, King Khalid University, Abha, Saudi Arabia

## Abstract

**Background:**

Assessment of cardiorespiratory fitness (CRF) is a standard procedure in routine clinical practices. Early identification of risk factors through screening is vital in the fight against chronic diseases. Evaluation of CRF can impose cost implications in the clinical setting; thus, a simple and easy-to-use test is to be advocated. The Ruffier test is a simple test that can assess CRF, and it is necessary to find whether the test reflects the effects of compounding factors in CRF.

**Objective:**

This study aims to determine the association between CRF (estimated VO_2max_) with cardiovascular, anthropometric, and physical risk factors using the Ruffier test.

**Methods:**

A cross-sectional study with a sample of 52 male participants was conducted. Before the Ruffier test, each participant's body weight, height, waist circumference, skinfold thickness, thigh length, lower-limb length, thigh circumference, physical activity, blood pressure, smoking, diabetes, and pulmonary functions were recorded, and these factors correlated with CRF.

**Results:**

There was a significant inverse relationship found between the estimated VO_2max_ and age, height, body weight, body mass index, waist circumference, a sum of skinfold, fat percentage, thigh length, lower-limb length, thigh circumference, smoking, blood pressure, heart rates, and diabetes (*p* < 0.05). A significant positive correlation was found between the estimated VO_2max_ with physical activity and respiratory functions (*p* < 0.05). In the multivariable model, body weight and resting heart rate were significantly inversely associated with the estimated VO_2max_(*p* < 0.05).

**Conclusion:**

Using the Ruffier test, various risk factors of CRF are correlated with the estimated VO_2max_. This test reflects the effects of different compounding factors on CRF; therefore, it can be used in routine clinical practices to identify the risk factors early.

## 1. Introduction

Dr. J. E. Ruffier introduced a test that measures the resistance of the heart to the physical effort [1]. The Ruffier test is simple, valid, easily reproducible, and requires no equipment except a timer. It has been popularly utilized in the fields of rehabilitation, physical education, and sports medicine and has been widely used for many years to measure the exercise performance in European countries [2–4]. It is a three-minute heart rate- (HR-) based cardiorespiratory fitness (CRF) test in which the participants perform 30 squats in 45 seconds. Three measurements of HR are taken: resting HR (pretest), HR immediately after completing the squats (post-test 1), and recovery HR 60 seconds, which is measured after the completion of the test (post-test 2). Studies have proven that the test has strong validity to predict CRF in terms of the estimated maximal oxygen uptake (VO_2max_) [5, 6].

VO_2max_ is defined as the maximal amount of oxygen consumption beyond which no further increase in oxygen consumption occurs with a further increase in exercise intensity [7]. VO_2max_ is a well-known and reliable method for quantitatively measuring CRF. CRF reflects the integrated ability to transport oxygen from the atmosphere to the mitochondria to perform physical work, and it quantifies the functional capacity of an individual. The measurement of CRF can be performed directly or can be estimated using the maximal work rate achieved on a treadmill or a cycle ergometer or from other simple or complex methods [8]. Various types of equipment, advanced labs, high cost, or bigger space are required to administer the available tests which measure CRF [9]. Thus, an alternative test is needed, which is valid and administered with ease.

Disease risk prediction is closely associated with CRF in apparently healthy individuals, individuals with risk, and individuals already diagnosed with one or more chronic conditions [10–14]. There are various proven relationships between CRF and respiratory functions [15–19], physiological [20], and physical factors [21, 22], smoking [23, 24], physical activity [25–28], and anthropometric variables [29]. Factors such as body weight, body mass index (BMI), waist circumference, fat percentage, blood pressure (BP), smoking, and diabetes inversely affect CRF, while respiratory functions and physical activity positively affect CRF.

A valid and reliable test not only measures the variables accurately but also should reflect the various compounding factors which have a positive or negative effect. Therefore, the standard tests can be administered to all irrespective of the influence of any compounding factors. In general, administering the screening tool such as the Ruffier test must help to measure the variables, as well as find out the influencing risk factors which cause major health issues. Hence, these screening tests assist in early identification and primary prevention of chronic diseases.

The relationship between CRF and its various risk factors have not been investigated using the Ruffier test. Therefore, this present study aimed to determine the correlation between CRF and cardiorespiratory, anthropometric, and physical risk factors using the Ruffier test. The second aim is to compare the difference in CRF between smoking and nonsmoking groups and between participants with and without diabetes.

## 2. Materials and Methods

In this cross-sectional study, the participants, including 52 male volunteers between 20 and 60 years of age (34.4 ± 2.4), were randomly selected from the student and faculty registry of University (male section) using a systematic random sampling method. The sampling interval was calculated using formula (*K*=*N*/Tsz). *N* denoted the total number of students and faculties and was 998, and Tsz is the total sample size. So, every 19^th^ volunteer was selected from the registry. A routine medical examination of the neuromusculoskeletal system was performed on the participants to determine their health status, and participants were requested to complete the Physical Activity Readiness Questionnaire [30]. Those found to be clinically healthy were requested to complete the informed consent form. Before signing the form, the participants received a complete verbal description of the benefits and risks of the study. Potential participants with self-reported acute infections, heart and lung diseases, a recent injury in the lower limbs, or neurological and cognitive disease were excluded. This study approved by the Research Ethics Committee, King Khalid University, Saudi Arabia (REC # 2018-06-02).

### 2.1. Measurement Procedures

**Figure 1:**
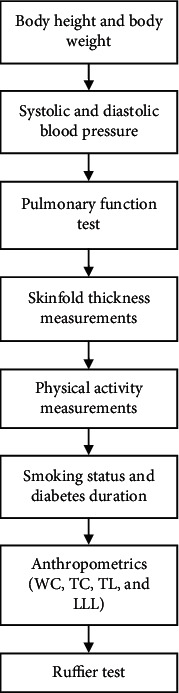
Sequence of measurement procedures. WC- waist circumference, TC- thigh circumference, TL- thigh length, and LLL- lower-limb length.

The sequence of measurement procedures is illustrated in Figure 1. Standing height (cm) and barefoot body weight (kg) were measured by using a stadiometer (Jiangsu Suhong, China) and a standard weighing machine (Joycare, China), respectively, while the participant wore a minimal amount of clothing. The BMI formula (body weight/height in meters^2^) was used to determine the BMI. The systolic blood pressure (SBP) and diastolic blood pressure (DBP) were measured in the seated position using an electronic sphygmomanometer (OMRON M7, Intelli IT, China).

Skinfold thickness (SFT) was measured by a single trained researcher using a Harpenden Caliper (Baty, UK) from carefully marked sites on the biceps, triceps, subscapular, and suprailiac areas on the nondominant side. The calipers were calibrated for tension and with a substance of known width before testing. Sites were carefully marked, and a minimum of two readings were taken at rotating sites; if two measures at a site differed by more than 3 mm, a third measure was taken. The mean of the two closest measures recorded and the percentage of body fat were calculated using the table published by Durnin and Womersley [31].

The anthropometric measurements were performed as follows: waist circumference (WC) in cm was measured using plastic tape at the midpoint between the costal margin and iliac crest in the midaxillary line in the standing position at the end of a gentle expiration [32]. Measurement sites were marked with a semipermanent ink pen and maintained throughout the experimental period. The thigh circumference (cm) was measured using a tape measured at 30, 50, and 70% of the distance from the greater trochanter point to the lateral condyle, using a pen marker to point to the place while the subject was standing upright [33]. Thigh length (cm) was measured between the greater trochanter and the lateral femoral condyle [34]. Lower-limb length was measured in a supine position from the most inferior aspect of the anterior superior iliac spine to the most distal aspect of the medial malleoli [35].

To quantify the participants' physical activity, they answered the short version of the International Physical Activity Questionnaire (IPAQ) [36]. The short version of the IPAQ addresses the number of days and minutes spent performing physical exercise in the form of recreational and occupational activities, transportation, and household duties. The score was obtained by totaling the number of days, hours, and minutes of physical activities performed during the week before completion of the questionnaire. The participants' smoking status was quantified using the Fagerstrom Test for Nicotine Dependency [37], which consists of six items.

The respiratory parameters were measured using the MIR Spirolab III (Italy) spirometer instrument. The subjects sat on a chair in a comfortable position and carefully fitted with the spirometer mouthpiece. The pegs were fitted to the nose to prevent air leakage. The procedure of lung function measurement consisted of forced vital capacity (FVC), forced expiratory volume/one second (FEV1), and the (FEV1/FVC) ratio. Measurements were repeated three times at five-minute intervals, and the highest score was selected for analysis.

The Ruffier test was performed at the end of all measurement procedures. Participants rested for five minutes in the supine position. The pretest heart rate (HR 1) was measured in the standing position after the five-minute rest period. The subjects were, then, instructed to perform 30 squats in 45 seconds, with a tempo set by a metronome (80 beats per minute). Each repetition consisted of two movements: squatting down and standing back up. The squatting movements were composed of flexion of the knee to 90° while keeping the back straight and the arms extended frontally. At the end of the test, the post-test heart rate 1 (HR 2) was measured after 15 seconds of the test. The post-test heart rate 2 (HR 3) was measured after one minute of the test, and the HR was measured using a Polar heart rate monitor (POLAR T31, China) [5, 6]. The VO_2max_ was estimated based on the following equation [5]:

(1) $$\begin{array}{c} VO_{\mathrm{2max}}=3.0143+1.1585\times \mathrm{sex}-0.0268\times \left(\frac{P1}{\mathrm{height}}\right)+118.7611\times \left[\frac{\left(P2-P3\right)}{\mathrm{ag}e^{3}}\right]. \end{array}$$

### 2.2. Statistical Analysis

Statistical software SPSS (version 20.0, IBM-SPSS Inc, Armonk, NY, USA) was used for statistical analysis. The type of data was checked by the Shapiro–Wilk test and found not distributed normally. The results were reported as mean ± SD, and the relationship between variables was determined using the Spearman correlation coefficient (*r*). Multiple linear regression was performed to examine bivariate correlations between the estimated VO_2max_ and compounding factors. The difference between groups was analyzed using the Mann–Whitney *U*-test. *p* < 0.05 was considered to be statistically significant.

## 3. Results

The study involved 52 healthy and asymptomatic participants. The participants' baseline characteristics are summarized in Table 1. Subjects with a history of smoking (18 participants, 34.6%) and diabetes (15 participants, 28.8%) also participated in the study. A significant inverse relationship was found between the estimated VO_2max_ and body weight (*p* ≤ 0.001), BMI (*p* ≤ 0.001), WC (*p* ≤ 0.001), the sum of skinfold (*p* ≤ 0.001), fat percentage (*p* ≤ 0.001), and diabetes duration (*p*=0.03). A significant inverse relationship was found between the estimated VO_2max_ and age (*p*=0.01), height (*p*=0.03), thigh length (*p*=0.02), lower-limb length (*p*=0.048), thigh circumference (*p*=0.02), smoking (*p*=0.02), SBP (*p*=0.03), DBP (*p*=0.02), resting heart rate (HR 1) (*p*=0.01), peak heart rate (HR 2) (*p*=0.02), and recovery heart rate (HR 3) (*p*=0.02).

**Figure 2:**
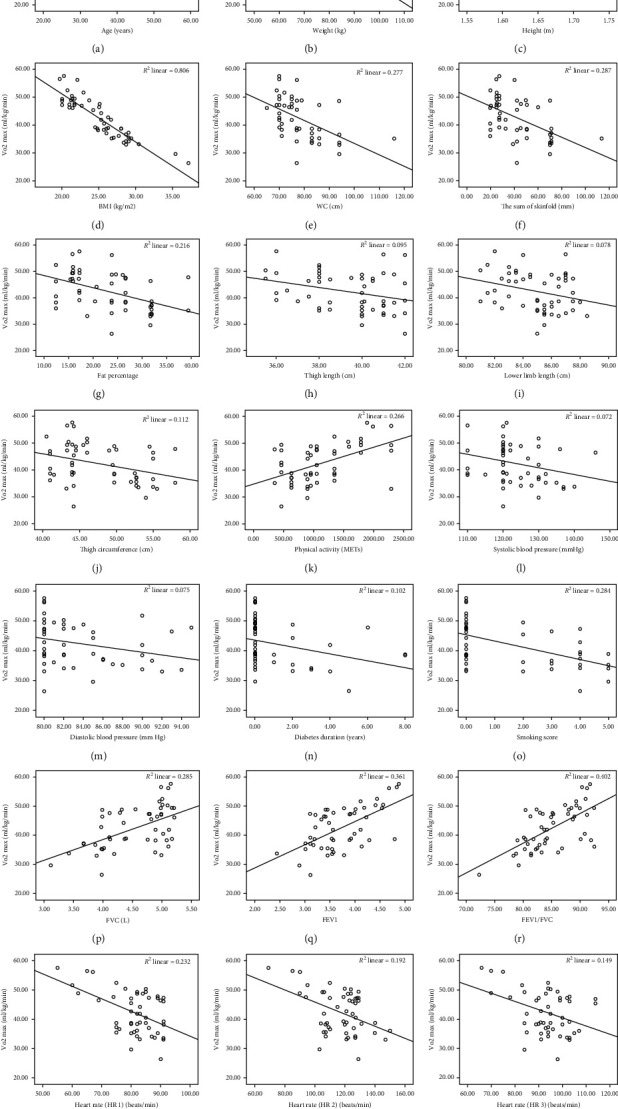
The relationship between the estimated VO_2max_ and (a) age, (b) weight, (c) height, (d) body mass index, (e) waist circumference, (f) the sum of skinfold, (g) fat percentage, (h) thigh length, (i) lower-limb length, (j) thigh circumference, (k) physical activity, and (l) systolic blood pressure. The relationship between the estimated VO_2max_ and (m) diastolic blood pressure, (n) diabetes duration, (o) smoking, (p) forced vital capacity, (q) forced expiratory volume in the first second, (r)(FEV1/FVC), (s) pre-exercise heart rate (HR 1), (t) postexercise heart rate (HR 2), and (u) postexercise heat rate (HR 3).

A significant positive correlation (*p* < 0.01) was found between the estimated VO_2max_ with physical activity (*p* ≤ 0.001) and respiratory functions (FVC, FEV1, and (FEV1/FVC)) (*p* ≤ 0.001) (Table 2). The differences between smokers and nonsmokers and participants with and without diabetes are also given in Table 2. A statistically significant difference was found between these groups. In the multivariable model (Table 3), the compounding factors of body weight, pr-test heart rate, and (FEV1/FVC) are significantly inversely associated with the estimated VO_2max_. The relationships between the estimated VO_2max_ and all other variables are illustrated in Figure 2.

## 4. Discussion

Assessment of CRF is vital for patients who are at risk of developing the disease in the cardiorespiratory system, as well as for athletes and the general public. The assessment of CRF using the Ruffier test is a known and valid method in various clinical settings [5]. An accurate test should reflect in its results the various positive and negative factors which affect the condition. There are various known risk factors and etiological factors that may lead to the development of the cardiorespiratory disease. Thus, the aim was to use the Ruffier test to assess the correlation between CRF and various risk factors for CRF.

Studies [38–42] report that VO_2max_ depends on age, gender, physical activity, and body weight. Maximal exercise capacity or VO_2max_ declines 5–20% per decade among healthy individuals with decreasing muscle mass and declining age-related physical activity levels [43]. Various authors conclude that there is an inverse low-to-moderate relationship between VO_2max_ and age. Similarly, the current study also reports a significant low correlation (*r* = −0.352, *p* < 0.05) between these variables.

In this study, height also reported a significant low inverse correlation (*r* = −0.310, *p* < 0.05) with the estimated VO_2max_. Previous studies have also reported mixed results, with height both correlating [44–48] and not correlating [49–51] with VO_2max_. The participants' performance of a simple knee flexion-to-extension movement in this test could explain the negative correlation since the other studies did not use this testing method. The body weight, BMI, and WC exhibit a moderate-to-very high negative correlation with estimated VO_2max_ using the Ruffier test (Table 2). Barry et al. [52] and Montero D and Diaz-canestro [53] concluded in their meta-analysis and systematic analysis, respectively, that there is an inverse relationship between BMI and CRF. Studies have also established inverse relationships between VO_2max_ and BMI and VO_2max_ and body weight, among students [54], athletes [55], and healthy adults [56]. A research [57] has indicated that a higher WC and lower maximal oxygen consumption produce results similar to this, and the present study also reports the same. Increasing type II muscle fibers and decreasing type I muscle fibers are one reason for decreasing VO_2max_ among high BMI and WC individuals [58].

Estimation of the body fat percentage using a skinfold thickness measurement is a reliable method across all age groups [59]. The current study reports a negative correlation for both the sum of skinfold thickness and body fat percentage (Table 2) with the estimated VO_2max_. These findings well supported in previous studies conducted by Drake et al. [60] and Montero and Diaz-Canestro [53]. The reason for the negative correlation between VO_2max_ and body fat is that there is a direct link between skeletal muscle mass and its capacity for generating oxygen and/or consuming oxygen [61]. The Ruffier test performed using the lower limbs, especially the thigh and its musculature. Thus, the relationship between outcomes of the Ruffier test examined with lower-limb variables such as limb length, thigh length, and thigh circumference. The present study reports a significant low negative correlation between the estimated VO_2max_ and lower-limb variables. The total body height, lower-limb length, and thigh length are negatively correlated with the estimated VO_2max_ using the Ruffier test.

Regarding thigh circumference, individuals with high BMI and body fat percentage may have high thigh circumference. This may explain the significant low negative correlation between thigh circumference and estimated VO_2max_, which was also reported in a study by Ko et al. [62], including college students as participants. Authors [63] also found a positive correlation between thigh circumference and CRF. To further investigate this relationship, researchers can perform research using real-time ultrasound measurement of thigh musculature and its relationship with VO_2max_.

A systematic review conducted by Echouffo-Tcheugui et al. [64] found a positive relationship between physical activity and CRF. The present study also reports a highly significant positive correlation between these variables (Table 2). Physical activity or exercise increases VO_2max_ by enhancing the cardiac output and the secondary to high stroke volume and causing an increase in arteriovenous oxygen difference. All these processes improve the extraction of oxygen by working muscle [65]. The present study also aimed to evaluate the relationship between the estimated VO_2max_ and diabetes, as well as smoking. The results revealed a negative correlation between these factors (Table 2). Systematic reviews [66, 67] and articles [23, 24] also establish an inverse relationship between CRF and diabetes, as well as smoking. Significant differences in the mean score (Table 2) were found in this study when analyzing the differences between smokers and nonsmokers and participants with and without diabetes. Impairments in nutritive blood flow to working muscles and endothelial-specific impairments are the causes of decreased exercise capacity among individuals with diabetes [68]. Smoking leads to the elevation of carbon monoxide and nicotine in the blood, which decreases the oxygen-carrying capacity of blood and causes the same decline in cardiorespiratory fitness among smokers [69].

Studies confirmed the negative relationship between BP and VO_2max_ [70, 71]. The present study also reports a significantly low inverse correlation between BP and the estimated VO_2max_ (Table 2). Increased BP leads to arterial stiffness and decreases the ability to transport blood to the working muscle [72]. The relationship between the pulmonary function and estimated VO_2max_ was analyzed in the present research. Various studies [15–17] conclude that there is a positive association between the pulmonary function (FVC, FEV1, and (FEV1/FVC)) and VO_2max_. The present study also found a highly significant moderate positive correlation between the pulmonary function and estimated VO_2max_. Better functioning of respiratory muscles, a favorable change in chest wall mechanics, and improved lung or airway perfusion are the mechanisms behind the positive correlation between lung function and VO_2max_ [73]. Studies using the Ruffier test [5, 6] to predict VO_2max_ showed the inverse relationship between three different heart rates (pretest heart rate [HR 1], post-test heart rate (HR 2), and post-test heart rate (HR 3)) and the estimated VO_2max_. The current study also established a negative correlation between HR and the estimated VO_2max_ (Table 2). Endurance activities generally increase the VO_2max_, and individuals with suitable endurance activities have low sympathetic activity on the conductive cardiac system [74], which leads to decreased HR among individuals with high VO_2_ max. This study further shows that the VO_2_ max estimated by the Ruffier test is inversely correlated with body weight and resting heart rate. In the multivariable model incorporating the individual compounding factors, body weight and resting heart rate are independently associated with the estimated VO_2_ max. These findings are consistent with previous studies those examined the influence of body weight [57, 75] and resting heart rate [76, 77] on VO_2max_.

The present study also reports a few limitations. The participants of this study have several residual fitness statuses, making it difficult to generalize the result on a specific uniform population. Other cardiorespiratory risk factors, such as biochemical variables and genetic factors, were not included. Furthermore, only males participated in this study due to the cultural norms of the country where it took place. The subjective assessment of physical activity was performed in this study; future studies may seek to obtain the objective assessment of physical activity.

## 5. Conclusions

The various proven risk factors of cardiorespiratory functions are positively or negatively correlated with the estimated VO_2max_ when measured using the Ruffier test. This test results reflect the different compounding factors of the cardiorespiratory system, which increase or decrease in the CRF. Hence, the Ruffier test may be administered to the general population to find the presence of compounding factors that affects the cardiorespiratory system at the earliest. Therefore, this test places a significant role in preventive care.

## Figures and Tables

**Table 1:** Basic characteristics of participants.

Variables	Mean ± SD	Minimum	Maximum
Age (yrs)	36.38 ± 10.49	21	57
Weight (kg)	71.67 ± 11.89	56	105
Height (m)	1.69 ± 0.39	1.59	1.75
BMI	25.12 ± 3.83	19.71	37.2
WC (cm)	78.4 ± 9.37	65	116
Sum of SF	43.54 ± 21.14	19.7	113.6
Fat percentage	23.19 ± 7.42	12.4	39.4
Thigh length (cm)	39.34 ± 1.93	35.5	42
LLL (cm)	84.92 ± 2.01	81	88.5
TC (cm)	47.63 ± 5.16	40.5	58
Physical activity (METs)	1114 ± 549.65	347	2292
SBP	123.56 ± 7.85	110	146
DBP	83.62 ± 4.41	80	95
Smoking score	1.38 ± 1.88	0	5
Diabetes (yrs)	1.02 ± 2.01	0	8
FVC (L)	4.55 ± 0.55	3.11	5.21
FEV1 (L)	3.71 ± 0.55	2.44	4.87
(FEV1/FVC)	85.07 ± 4.54	72.31	92.48
HR 1	81.09 ± 8.28	55	91
HR 2	116.85 ± 15.46	69	150
HR 3	93.04 ± 10.02	66	114
Estimated VO_2max_	42.39 ± 7.31	26.43	57.57

BMI: body mass index; WC: waist circumference; SF: skinfolds; LLL: lower-limb length; TC: thigh circumference; SBP: systolic blood pressure; DBP: diastolic blood pressure; FVC: forced vital capacity; FEV1: forced expiratory volume in 1 second; HR: heart rate; VO_2max_: maximal oxygen consumption.

**Table 2:** Correlation between the estimated VO_2max_ and various compounding factors of CRF. Difference in the estimated VO_2max_ among smokers vs. nonsmokers and diabetes vs. nondiabetes.

	Age	BW	Ht	BMI	WC	Sum of SF	BF	TL	LLL	TC	PA	DD	Smoking
Estimated VO_2max_	−0.352 *p* < 0.05	−0.910 *p* < 0.01	−0.310 *p* < 0.05	−0.897 *p* < 0.01	−0.607 *p* < 0.01	−0.549 *p* < 0.01	−0.496 *p* < 0.01	− 0.316 *p* < 0.05	−0.276 *p* < 0.05	−0.327 *p* < 0.05	0.478 *p* < 0.01	−0.406 *p* < 0.01	−0.514 *p* < 0.05

	SBP	DBP	FVC	FEV1	(FEV1/FVC)	Pretest heart rate (HR 1)	Post-test heart rate (HR 2)	Post-test heart rate (HR 3)	Smokers Mean ± SD	Nonsmokers Mean ± SD	Diabetes Mean ± SD	Non-diabetes Mean ± SD	
Estimated VO_2max_	−0.307 *p* < 0.05	−0.324 *p* < 0.05	0.523 *p* < 0.01	0.559 *p* < 0.01	0.597 *p* < 0.01	−0.343 *p* < 0.05	−0.315 *p* < 0.05	−0.330 *p* < 0.05	37.76 ± 5.94	45.29 ± 6.62	37.69 ± 5.96	44.29 ± 6.99	
									*T* = 4.14, *p* < 0.01		*T* = 3.21, *p* < 0.01		

VO_2max_: maximal oxygen consumption; BW: body weight; Ht: height; BMI: body mass index; WC: waist circumference; SF: skinfolds; BF: body fat percentage; TL: thigh length; LLL: lower-limb length; TC: thigh circumference; PA: physical activity; DD: diabetes duration; SBP: systolic blood pressure; DBP: diastolic blood pressure; FVC: forced vital capacity; FEV1: forced expiratory volume in 1 second; HR: heart rate.

**Table 3:** A multivariate model of association of the estimated VO_2max_ with compounding factors.

	Beta coefficient	Adjusted *R*-squared = 0.42		
		95% CI		*p* value
Bodyweight (kg)	−0.56	−0.63	−0.49	<0.001
Pre-test heart rate (HR 1)	−0.32	−0.36	−0.27	<0.001
Physical activity	0.001	0.000	0.002	<0.05
(FEV1/FVC)	−0.12	−0.23	−0.007	<0.05

95% CI: 95% confidence interval; HR: heart rate.

## Data Availability

The data used to support the findings of this study are available from the corresponding author upon request.
